# Impact of health science popularization videos on user perceived value and continuous usage intention: based on the C-A-C and ECM model framework

**DOI:** 10.3389/fpubh.2024.1382687

**Published:** 2024-07-01

**Authors:** Wenxia Xuan, Thanawan Phongsatha, Lijie Hao, Kun Tian

**Affiliations:** ^1^Shanxi Bethune Hospital, Shanxi Academy of Medical Sciences, Tongji Shanxi Hospital, Third Hospital of Shanxi Medical University, Taiyuan, China; ^2^Graduate School of Business and Advanced Technology Management, Assumption University, Bangkok, Thailand; ^3^Department of Curriculum and Instructional Technology, Faculty of Education, Universiti Malaya, Kuala Lumpur, Malaysia; ^4^College of Digital Arts, Communication University of Shanxi, Taiyuan, China

**Keywords:** health science popularization videos, cognitive-affective-conative, expectation-confirmation model, Chinese residents, perceived value, affective, continuous usage intention

## Abstract

**Objective:**

To enhance individuals’ sustained intention to use health science popularization videos, this study investigated the path relationships and influencing mechanisms of health science popularization video factors on users’ perceived value, expectancy confirmation, enjoyment, satisfaction, trust, and continuous usage intention based on the cognitive-affective-conative and expectation-confirmation model theoretical framework.

**Methods:**

This study adopted a cross-sectional design and collected data using self-administered questionnaires. The hypotheses were analyzed using the smart partial least squares (Smart-PLS) structural equation modeling method with a dataset containing 503 valid responses. Subsequently, comprehensive data analysis was conducted.

**Results:**

Blogger and video quality factors present in health science popularization videos substantially influenced users’ perceived value (*p* < 0.001). Furthermore, users’ expectancy confirmation exerted a positive influence on both users’ perceived value (*p* < 0.001) and satisfaction (*p* < 0.01). Perceived value, in turn, positively impacted satisfaction (*p* < 0.001) and pleasure (*p* < 0.001). User satisfaction (*p* < 0.001) and pleasure (*p* < 0.001) directly enhanced trust, which, in turn, significantly and directly impacted continuous usage intention (*p* < 0.001).

**Discussion:**

This study offers both theoretical and practical insights into enhancing the quality of health science popularization videos. From a theoretical perspective, it expands upon the cognitive-affective-conative and expectation-confirmation model theoretical frameworks, enriches the theoretical model, and offers theoretical references for future research in this domain. From a practical perspective, enhancing the overall quality of health science popularization content significantly influences users’ perceived value, emotional engagement, and continued usage intention to engage with the content.

## Introduction

Health literacy refers to an individual’s ability to accurately utilize health information and services to uphold and advance well-being. It not only signifies the level of development within a nation and society but also holds significance at the personal level. According to 2021 data from the Chinese National Health Literacy Surveillance, the overall health literacy level among residents was 25.40%. Within this, health literacy accounts for 35.93%, chronic disease prevention and management occupies 26.67%, and basic medical literacy accounts for 26.05% of the overall level. This overall level remains modest, and a notable discrepancy exists between developed and underdeveloped regions (1, 2). To effectively heighten the populace’s health literacy, the Chinese government has proactively implemented responsive measures and introduced corresponding policies, such as “Healthy China 2030” (3) and “Healthy China Action” (4). Despite discernible progress resulting from these endeavors, residents’ collective health literacy falls short of the desired standard (5). Consequently, the endeavor to effectively elevate the health literacy level of the population is a focal concern for the Chinese government and various segments of society.

In the current Internet era, health information has become an integral aspect of people’s lives. It not only shapes their daily routines but also significantly influences their health literacy levels (6, 7). Against this backdrop, issues related to health literacy have garnered substantial attention in the academic community. Concerning health information, research has predominantly focused on evaluating the quality of online health information and investigating the mechanisms underlying user interactions, trust, and willingness to use health-related information (8–10). Furthermore, some studies have regarded health information as a complementary element to medical interventions, playing a pivotal role in aiding patient recovery and psychological well-being (11, 12). In the realm of health literacy, scholars have explored the role of the Internet in health information dissemination, as well as the avenues and mechanisms through which it enhances public health literacy and awareness (13, 14). Concurrently, some researchers have observed that offline activities (community events, popular science lectures, etc.), as supplements to online health science popularization information dissemination, play a pivotal role in elevating public health literacy (15). While scholars have extensively researched improving the quality of health information and public health literacy from various perspectives, studies on the relationship between health science videos and users’ psychological activities are relatively scarce. In particular, an increasing number of mobile social platforms (such as WeChat, TikTok, and Xiaohongshu) have become vehicles for disseminating health science information, making it easy for people to access and utilize such information through these platforms. Consequently, among the health science popularization videos, what types of content and formats can engender users’ perceived value? Do these perceived values meet their expectations? Furthermore, are video content and format key factors influencing users’ emotional states and continuous use of health science popularization videos? Investigating these questions is crucial for the future development of health science popularization videos and holds the key to effectively enhancing individuals’ health literacy and health information quality.

Presently, video-based health science popularization information has emerged as a predominant medium that enables individuals to establish effective interactive and communicative relationships with health information (16). Within this interactive process, individuals seek relevant health science popularization videos based on their specific needs. Additionally, the content and presentation style of health science popularization videos also impact individuals’ anticipations and emotional states and subsequently influence their subsequent behavioral intentions (17, 18). Prior research has indicated that the cognitive-affective-conative (C-A-C) model effectively verifies the causal relationships between users’ information cognition, emotions, and behaviors (19, 20). Similarly, the expectation-confirmation model (ECM) aptly illustrates the positive impact of perceived value from expectation confirmation on user satisfaction (21). Both theoretical models serve as robust tools for exploring and validating connections between users and information. Consequently, this study aims to extend the theoretical frameworks of C-A-C and ECM while integrating past literature to investigate how latent factors within the content and format of health science popularization videos influence users’ perceived value, expectation confirmation, emotions, and subsequent sustained usage behavior. The findings of this study will elucidate how the content and format of short health science popularization videos influence users’ psychological activities and how these complex psychological changes ultimately impact their willingness for continued use. This not only extends and enriches existing research frameworks but also fills in some knowledge gaps in the field of health science video dissemination. Furthermore, it provides theoretical and practical guidance for the dissemination and design of health popularization science videos, as well as for the development of related fields.

The remaining structural components of this study are as follows: the literature review and hypothesis development sections introduce the C-A-C and ECM, and the hypotheses are formulated based on these models. The research methodology section outlines the survey design, target population, and data collection procedures. The results section presents the findings of the survey and model outcomes. The discussion section interprets the research findings. Finally, the paper concludes with implications, limitations, and conclusions.

## Literature review and hypotheses development

### C-A-C

Typically, human behavior follows a systematic and purposeful pattern. Prior to decision-making, individuals frequently engage in contemplation and planning (22). Within the C-A-C model, the interplay and influence between individuals’ cognition and emotions in the course of an activity are well elucidated, along with how this relationship culminates in the eventual occurrence of behavior (23). For instance, the process of consumer behavior entails three stages: cognitive (thinking), affective (feeling), and conative (doing). When consumers engage in purchasing goods, they actively seek information about the products (advertisements, store descriptions, etc.). This product information, in turn, influences their anticipations and emotional shifts, ultimately steering their purchasing decisions (24). Over time, the C-A-C framework continuously validates the process of individuals’ cognition, emotions, and behavior. Numerous scholars have expanded upon the C-A-C theory to encompass related theoretical models, such as the ECM (21), the Technology Acceptance Model (TAM) (25), and the American Customer Satisfaction Index Model (26). These expansions seek to elucidate the relationships among cognition, emotions, and behavior across various life contexts. Numerous studies have confirmed the applicability of the C-A-C framework in diverse industry domains. For instance, in the realms of business and service, the model verifies the interplay between consumer-perceived brand value and consumption behavior (27). The psychological and consumption impacts of IT product factors on consumers have been underscored (28). Perceived value, attitudes (satisfaction and trust), and their influences on consumer purchasing intentions have been explored in the context of tourist destinations (29). In the domains of online information and social interactions, the C-A-C framework has similarly demonstrated associations between users’ perceived value, loyalty, and continuous usage intentions toward information (30).

Building upon these foundations, this study adopts the C-A-C framework as its base theoretical foundation to construct a theoretical framework that delineates the sustained usage intentions of users toward health science popularization videos. This framework aims to illuminate the inherent mechanisms linking the content and format of health science popularization videos with users’ perceived value and their intentions for continued usage.

### ECM

The expectation confirmation theory (ECT) was originally employed to scrutinize and comprehend consumer behavior, effectively delving into the outcomes arising from the alignment of consumers’ pre-purchase expectations with post-purchase performance. It delineates how this alignment influences the relationship between consumer satisfaction and consumption behavior (31). As a result, the theory has found extensive application in diverse marketing domains, including the fast-moving consumer goods industry (32, 33) and service sector (34, 35). As the theory extended its scope to the realm of information systems, the ECM was formulated and applied to explore users’ intentions for continued usage of information system products/services (36, 37).

The ECM is a useful approach for examining intricate information systems, especially for understanding how new factors impact users. It emphasizes the perceived utility following the utilization of products/services and aligns with the degree of expectation confirmation. This alignment facilitates a more comprehensive exploration of the relationships between users and information systems (36, 38). While the ECM effectively analyzes the impact of factors such as information quality and service quality on users’ perceptions and their relationship with usage intentions, it lacks an analysis of users’ perceived value and emotional shifts following the usage of information system products/services, as well as the subsequent impact on sustained usage intentions (36, 38). However, the C-A-C framework can effectively predict users’ perceived value and emotional shifts following product usage. Thus, in this study, we adopted the C-A-C theory as a support framework for the ECM, enhancing the construction of a relationship model framework between users and health science popularization videos.

The value of media information can be classified into two categories: utilitarian and hedonic. Utilitarian value refers to the practical utility of the information content, whereas hedonic value represents the impact of the information content on users’ mental and emotional states (39, 40). During the process of using media information, individuals’ expectation confirmation is often influenced by the content and format of the information, as well as the perceived value post-usage. These influences subsequently contribute to the generation of subsequent sustained behaviors (41, 42). Similarly, health science popularization videos, as a type of media information, possess the same value attributes as media information. For instance, when individuals have health-related needs, they actively seek out and engage in health science popularization videos to obtain health information, accompanied by their expectations. In this process, the ability of the information content and format to evoke perceived value and whether this perceived value aligns with their expectations before searching not only impact individuals’ emotional shifts, satisfaction, and trustworthiness but also holds paramount significance in determining whether they develop a sustained intention to use.

Building on the C-A-C model and incorporating the ECM, this study constructs a novel theoretical framework that comprehensively investigates how the content and format of health science popularization videos influence individuals’ perceived value, expectation confirmation, emotional shifts, satisfaction, trustworthiness, and intention for sustained usage. Simultaneously, it unveils the interplay among these factors. This endeavor not only provides theoretical and practical guidance for the enhancement and development of existing health science popularization videos but also exerts a subtle yet influential role in elevating individuals’ health literacy levels in their everyday lives.

### Hypothesis development

Health science popularization videos constitute a comprehensive means of conveying health information, encompassing factors such as health information content, professionalism, information presentation, and interactive communication (43). These factors are manifested through the professional articulation of health bloggers, presentation of information content, and interactive communication within health science popularization videos. During this process, individuals acquire health knowledge by consuming video content, which influences their perceived value and intention for sustained usage. Therefore, in this study, the image, professionalism, information quality, and interactive quality of health bloggers were consolidated as critical factors that impact users’ perceived value.

#### Relationship between the appearance of health science popularization video bloggers and perceived value

Health bloggers play a crucial role in making complex health information more accessible to the public through explanations provided in health science popularization videos. They help bridge the gap between technical jargon and layperson comprehension, making the information more understandable, acceptable, and shareable. This effective dissemination of health information relies on bloggers’ capacity to bridge the gap between technical jargon and layperson comprehension (44). Existing research suggests that characteristics related to video bloggers’ celebrity status and appearance can influence users’ perceived evaluations and motivations for using products (45). Liu et al. (46) found that videos are better at presenting both the blogger’s image and information content than text or images, thus enhancing users’ emotional engagement and usage experience. Therefore, within the context of health science popularization videos, bloggers, as agents of health information dissemination, have the potential to evoke users’ emotions through their attractive appearance, clear expression, and authoritative demeanor. This, in turn, fosters trust and identification with the blogger’s persona, thereby stimulating users’ willingness to engage. Based on these premises, this study proposes the following hypothesis:

*H1*: The appearance of health science popularization video bloggers significantly influences users’ perceived value.

#### Relationship between the professionalism of health science popularization video bloggers and perceived value

Professionalism represents a concentrated embodiment of abilities based on knowledge, skills, attitudes, and more, reflecting the comprehensive competencies of practitioners in their respective fields. Health science popularization videos encompass scientific and health knowledge and embody a high level of professional requirements. Such videos must possess reliability and accuracy to establish cognitive authority, enhance user conviction, and identify with the information content (47, 48). However, in health science popularization videos, professionalism extends beyond the information content and encompasses the professionalism of the health bloggers themselves (49). For instance, health bloggers with medical backgrounds are able to provide health information in a professional and authoritative manner, which helps build trust and credibility among their audiences. This can lead people to reference the content for reliable information. Similarly, authoritative health information diminishes user panic and anxiety, thereby fostering a positive emotional connection with health information (50). Thus, this study proposes the following hypothesis:

*H2*: The professionalism of health science popularization video bloggers significantly influences users’ perceived value.

#### Relationship between the quality of health science popularization video information and perceived value

Health science popularization videos represent a multimodal form of information presentation that encompasses elements such as text, images, personalities, and special effects (51). The design and editing techniques used to create these elements are carefully crafted to produce visually appealing representations that effectively communicate health science popularization information. These elements are meticulously crafted through design and editing techniques to create esthetically pleasing representations that efficiently convey health science popularization information. Prior research has indicated that an interface information design with high-quality esthetic attributes can effectively evoke user emotions and enhance their willingness to download and engage (52). Furthermore, Chen et al. (53) have found that factors such as video duration, title, interactive loops, and content quality significantly impact user emotions and engagement levels. For health science popularization videos, the quality of information content is pivotal in disseminating health-related information; the quality of content directly influences users’ emotional responses. Therefore, this study proposes the following hypothesis:

*H3*: The quality of health science popularization video information significantly influences users’ perceived value.

#### Relationship between the quality of interaction in health science popularization videos and perceived value

Health science popularization videos offer greater convenience and interactivity than static information. Users can search for, access, and comment on the video content anytime and anywhere, catering to their physical and mental needs, thus accelerating the rapid dissemination of health knowledge (16). Existing research has confirmed that video-based health information evokes strong engagement, immersion, and interactivity. User interactions with videos directly influence their perceived value, emotional state, and willingness to use (54). Similarly, a high-quality interactive experience not only enhances positive emotions and engagement but also boosts trustworthiness (55). Based on these considerations, the following hypothesis is proposed:

*H4*: The interaction quality of health science popularization videos significantly influences users’ perceived value.

#### Relationship between perceived value, pleasure, and satisfaction in health science popularization videos

Perceived value pertains to the holistic assessment of a product (or service) by its users, considering diverse dimensions. These dimensions predominantly encompass the product’s quality, emotional attributes, and social worth (56, 57). Specifically, perceived value represents a balance between perceived benefits and perceived costs (58). When users utilize a particular product or service, if the perceived benefits outweigh the perceived costs, their perceived value increases; conversely, it decreases. Numerous studies have also affirmed that perceived value not only influences user behavior but also impacts the outcomes of their actions. For instance, perceived value affects user satisfaction, loyalty, and behavioral intentions (59–61). Furthermore, it has an impact on a user’s emotional perception, as it can evoke emotional states ranging from positive emotions, such as pleasure and excitement, to negative emotions, such as disappointment and pessimism (62). Consequently, when users watch and engage with health science popularization videos, they form a perceived value that varies in degree, which, in turn, influences their level of satisfaction. Similarly, throughout this process, users’ emotions are continually influenced by their perceived value, leading to diverse emotional changes. Existing research has indicated that positive emotional states effectively enhance user satisfaction (63). Based on these premises, the following hypotheses are proposed:

*H5*: Perceived value of health science popularization videos significantly affects user satisfaction.

*H6*: Perceived value of health science popularization videos significantly influences user pleasure.

*H7*: User pleasure with health science popularization videos significantly impacts user satisfaction.

#### Relationship between expectation confirmation and perceived value and satisfaction in health science popularization videos

Expectation confirmation refers to the evaluation and feedback of users’ prior expectations after actual product (or service) usage (64). In the context of the ECM, expectation confirmation is positively correlated with perceived value and user satisfaction (36). When individuals seek information to fulfill their needs, they usually have certain anticipations. If users find that the information meets their anticipated expectations and caters to their needs during actual usage, they tend to feel satisfied and consider the information useful and trustworthy. Ambalov’s meta-analysis results (65) also confirm the relationship between expectation confirmation, perceived value, and satisfaction. Similarly, this relationship has been validated across diverse research domains such as shopping, banking, and mobile applications (66–68). Therefore, based on the above discussion, the following hypotheses are proposed:

*H8*: Expectation confirmation significantly influences perceived value in health science popularization videos.

*H9*: Expectation confirmation significantly influences user satisfaction in health science popularization videos.

#### Relationship among pleasure, satisfaction, trust, and continuous use intention of health science popularization videos

Pleasure is the tangible manifestation of users’ emotions after engaging with a product or service and plays an influential role in their decision-making process (69). On social media, content can have a direct impact on user emotions (70). As key points of emotional expression in user behavior processes, satisfaction and trust play important roles in determining user behavioral outcomes. Previous research has confirmed that pleasure effectively impacts satisfaction and trust (71, 72). Satisfaction is an emotional expression of users’ behavior process that influences their subsequent actions. According to the customer satisfaction theory, satisfaction directly affects users’ willingness to make purchases (73). Trust, which is a crucial factor in users’ information utilization process, significantly influences their continuous usage behavior (74). Similarly, scholars have explored the relationship between information content and user emotions from a data computation perspective by employing machine learning models, sentiment analysis, and other methods to validate the accuracy of information content in influencing user emotions (75, 76). Quality health science popularization videos can generate pleasure and satisfaction for users, which can lead to increased trust in the video content. When satisfaction and trust are consistently present, users are more likely to continue using the videos. Drawing on this discourse, the following hypotheses are formulated:

*H10*: Pleasure in health science popularization videos significantly influence trust.

*H11*: Satisfaction in health science popularization videos significantly influences trust.

*H12*: Satisfaction in health science popularization videos significantly influences the continuous use intention.

*H13*: Trust in health science popularization videos significantly influences the continuous use intention.

### Construction of the conceptual model

**Figure 1:**
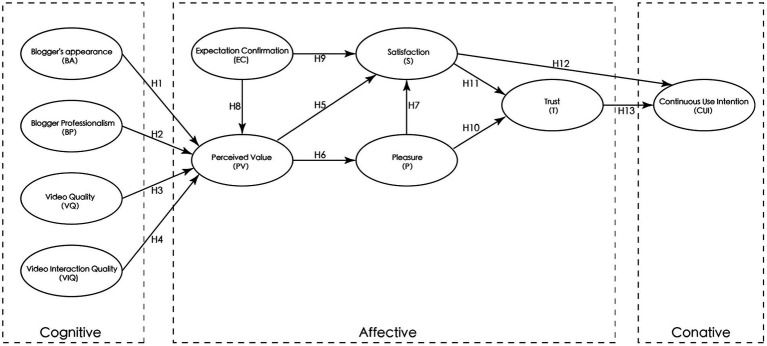
Model construction.

Based on the C-A-C and ECM models, a new model was built to explore the impact of health science popularization videos on users’ emotional states and willingness for continued use, as shown in Figure 1.

## Research method

### Sample selection and data collection

Based on the aforementioned hypotheses, this study designed a questionnaire and invited 10 participants with experience using health science popularization videos to complete a pilot-test. Ambiguous or easily misunderstood sections of the questionnaire were then revised to ensure the participants’ comprehensive understanding of the content and enhance the quality of the questionnaire. After completing the initial draft revisions, a pilot-test was conducted in a residential community in Taiyuan, China, resulting in the collection of 67 valid questionnaires. Following the reliability coefficient standards proposed by Nunnally and Bernstein (77), the Cronbach’s alpha value from this test exceeded 0.7, indicating acceptable internal consistency and stability of the questionnaire.

In terms of content validity, five experts with backgrounds in medicine and communication were invited to validate this questionnaire, which yielded an Index of Objective Consistency (IOC) of approximately 1, indicating that the content validity of the questionnaire was appropriately confirmed (78).

This study was approved by the Ethics Committee of Shanxi Bethune Hospital (YXLL-2022-021). The survey was conducted with the consent of each individual, and all resplendent data are kept confidential. The questionnaire was designed and produced on the Wenjuanxing (China) platform and distributed and collected using mobile devices. Online surveys offer several advantages (79): (1) Sampling is not limited to a solitary geographic area; (2) expenses are kept at a comparably low level; and (3) the survey boasts a swift response rate. To enhance participants’ engagement and trust, the instructions section of the questionnaire informed participants of the relevant requirements and explained that sincere completion of the questionnaire would lead to a random reward of no less than 10 RMB.

This study collected 560 surveys sourced exclusively from second- and third-tier Chinese cities. The collected data represent a reliable representation of urban dwellers in China (80). Table 1 outlines a detailed description of the questionnaire that was analyzed. A total of 57 responses were excluded during the data screening process due to incompleteness or a response duration of less than 5 min. Following thorough examination, 503 valid questionnaires were retained, resulting in a response rate of 89.8%.

**Table 1:** Demographic profile.

Measure	Items	Frequency	Percent
Gender	Male	239	47.51
	Female	264	52.48
Age	Below 18	65	12.92
	19–35	119	23.65
	36–50	157	31.21
	51–65	103	20.47
	Above 66	59	11.72
Education	Middle school and below	102	20.27
	High school	133	26.44
	Undergraduate	180	35.78
	Graduate and above	88	17.49
Income	Below 1,000	86	17.09
	1,001–3,000	93	18.48
	3,001–6,000	154	30.61
	6,001–10,000	101	20.07
	Above 10,000	69	13.71
Career	Student	94	18.68
	Private	117	23.26
	Public institution	142	28.23
	Civil servant	58	11.53
	Other	92	18.29

### Instruments

This study employed established scales from previous studies with appropriate modifications based on the research context. Regarding blogger images, reference was made to Ohanian’s study (81). The professionalism of bloggers was influenced by the study by Clyde et al. (82) Video quality and video interaction quality were drawn from the works of Lin and Wang (83), as well as Liu (84). The concept of perceived value was informed by the study Kim et al. (85) and Gupta and Kim (86) Expectation confirmation and satisfaction dimensions were based on the studies by Bhattacherjee (21), Bhattacherjee et al. (36), and Oliver (87). The measurement of pleasure was guided by the study by Watson et al. (88). Trust and intention to continue use were informed by the studies by Bhattacherjee (21), Bhattacherjee et al. (36), Davis et al. (89), and Bart et al. (90). The measurement elements were formulated utilizing a 5-point Likert scale, encompassing five gradations: “strongly disagree,” “disagree,” “undecided,” “agree,” and “strongly agree,” which were, respectively, assigned values of 1, 2, 3, 4, and 5. The questionnaire items are listed in Table 2.

**Table 2:** Variable scale and items.

Variable	Factor	Item	Reference
Blogger’s appearance (BA)	BA1	I find the blogger’s good appearance and posture pleasurable.	Ohanian (81)
	BA2	I find the blogger’s good temperament attractive.	
	BA3	I think the blogger’s formal attire gives me a sense of authority.	
	BA4	Overall, the blogger’s physical appearance is crucial in health popularization science videos.	
Blogger professionalism (BP)	BP1	The blogger’s performance in health popularization science videos is professional.	Clyde et al. (82)
	BP2	The blogger has affinity in health popularization science videos.	
	BP3	The blogger reflects the importance of professional ethics in health popularization science videos.	
Video quality (VQ)	VQ1	The presentation style of health popularization science videos can capture my attention.	Lin and Wang (83)
	VQ2	The content of health popularization science videos is comprehensive and meets my needs.	
	VQ3	The content of health popularization science videos is authentic and reliable, which makes me feel at ease.	
	VQ4	Health popularization science videos can link to relevant health information, allowing me to have a comprehensive understanding of health messages.	
Video interaction quality (VIQ)	VIQ1	I can inquire about health-related questions anytime through health popularization science videos.	Liu (84)
	VIQ2	Health popularization science videos can facilitate interaction between bloggers and users.	
	VIQ3	Health popularization science videos can offer health information that meets users’ needs.	
	VIQ4	Health popularization science videos provide users with more opportunities to seek health information.	
Perceived value (PV)	PV1	Health popularization science videos offer valuable content and services.	Kim et al. (85) and Gupta and Kim (86)
	PV2	Health popularization science videos provided by health education videos is practical to me.	
	PV3	Health popularization science videos provided by health education videos is beneficial to me.	
	PV4	Overall, Health popularization science videos provide substantial value.	
Expectation confirmation (EC)	EC1	The health popularization science videos I watched exceeded my expectations.	Bhattacherjee (21), Bhattacherjee et al. (36), and Oliver (87)
	EC2	The information, services, and features provided by health popularization science videos surpassed my expectations.	
	EC3	Overall, most of my expectations regarding the health popularization science videos I watched were confirmed.	
Pleasure (P)	P1	Watching health popularization science videos makes me feel (angry/happy).	Watson et al. (88)
	P2	Watching health popularization science videos makes you feel (unhappy/happy).	
	P3	Watching health popularization science videos makes you feel (painful/happy).	
Satisfaction (S)	S1	Using health popularization science videos makes me feel satisfied.	Bhattacherjee (21) and Bhattacherjee et al. (36)
	S2	Using health popularization science videos brings me a sense of enjoyment.	
	S3	Using health popularization science videos brings me great joy.	
	S4	Overall, health popularization science videos make me feel extremely satisfied.	
Trust (T)	T1	I trust that health popularization science videos can help me address the health concerns I have.	Davis et al. (89) and Bart et al. (90)
	T2	The information provided by health popularization science videos is helpful to me, and I have confidence in it.	
	T3	The content within the health popularization science videos information is trustworthy.	
Continuous use intention (CUI)	CUI1	I will continue to use health popularization science videos and will not switch to other alternatives.	Bhattacherjee (21) and Davis et al. (89)
	CUI2	I will continue to use health popularization science videos to acquire health knowledge and will not discontinue their use.	
	CUI3	I will maintain or even increase the frequency of using health popularization science videos.	

To uphold translation quality, we employed a procedure involving translation and subsequent back-translation, given that the data were gathered in China. As the primary step, we engaged two linguistic professors to evaluate the significance and comprehensibility of each item. Collaboratively, we proceeded to render the English questionnaire in Chinese with expert guidance. Subsequently, a doctoral student undertook the task of translating the Chinese questionnaire back to English. Ultimately, we meticulously scrutinized the translated elements against the original English rendition, refining the translation and rectifying any divergences to establish congruity between the two English iterations.

### Data analysis

In this study, the research hypothesis model was validated using the Smart-PLS. First, a comprehensive analysis of the reliability and validity of the model was conducted by estimating and validating the path coefficients and explanatory capacity of the structural model. These sequential processes aimed to substantiate the dependability and credibility of the model’s constructs, ultimately affirming their interconnectedness among these constructs (91). This method allowed us to investigate the causal relationships between variables and concurrently handle model construction and measurement items (92). Smart-PLS is not only lenient with respect to the normality and randomness of the sample but also suitable for addressing relationships among variables in datasets with non-normal distributions, and it possesses advantages in predicting complex predictive models (93). Majchrzak et al. (94) advised that a prudent guideline is to maintain a sample size that is at least 5–10 times the maximum number of model paths. In the context of this study, the sample size was 503, and the maximum count of path coefficients reached 13. This aligns with the conditions that render PLS a suitable choice for this analysis. For this study, the SmartPLS software (Version 3.3.7), developed by Ringle et al. (95), was employed.

## Findings

### Reliability and validity test

By analyzing the reliability of the variable items and the internal consistency, convergent validity, and discriminant validity of each construct, this study ensured that each item factor loading met the requirement of a threshold of 0.6 (96). The composite reliability (CR) for each construct exceeded the threshold of 0.7 (97), indicating a high degree of internal consistency within the constructs. Regarding convergent validity, the average variance extracted (AVE) index for each construct exceeded 0.5, suggesting satisfactory convergent validity for each construct (98), as shown in Table 3. As shown in Table 4, the square root of AVE for all variables was greater than that of the other variables, indicating good discriminant validity of the questionnaire.

**Table 3:** Model reliability and validity analysis.

Variable	Item	Standardized factor load	Cronbach’s alpha	CR	AVE
Blogger’s appearance (BA)	BA1	0.913	0.928	0.949	0.822
	BA2	0.909			
	BA3	0.910			
	BA4	0.894			
Blogger professionalism (BP)	BP1	0.925	0.907	0.942	0.844
	BP2	0.930			
	BP3	0.901			
Video quality (VQ)	VQ1	0.904	0.926	0.948	0.819
	VQ2	0.910			
	VQ3	0.903			
	VQ4	0.902			
Video interaction quality (VIQ)	VIQ1	0.887	0.918	0.942	0.802
	VIQ2	0.898			
	VIQ3	0.909			
	VIQ4	0.888			
Perceived value (PV)	PV1	0.875	0.906	0.934	0.780
	PV2	0.904			
	PV3	0.874			
	PV4	0.879			
Expectation confirmation (EC)	EC1	0.895	0.893	0.933	0.823
	EC2	0.918			
	EC3	0.909			
Pleasure (P)	P1	0.807	0.775	0.870	0.692
	P2	0.894			
	P3	0.790			
Satisfaction (S)	S1	0.871	0.903	0.932	0.775
	S2	0.890			
	S3	0.887			
	S4	0.874			
Trust (T)	T1	0.794	0.772	0.868	0.687
	T2	0.826			
	T3	0.866			
Continuous use intention (CUI)	CUI1	0.738	0.701	0.834	0.627
	CUI2	0.831			
	CUI3	0.804			

**Table 4:** Square AVE values and correlation coefficients of each variable.

	BA	BP	CUI	EC	P	PV	S	T	VIQ	VQ
BA	0.907									
BP	0.543	0.919								
CUI	0.473	0.581	0.792							
EC	0.646	0.677	0.702	0.907						
P	0.416	0.527	0.654	0.639	0.832					
PV	0.752	0.670	0.677	0.728	0.562	0.883				
S	0.477	0.649	0.778	0.673	0.767	0.680	0.881			
T	0.535	0.577	0.720	0.715	0.683	0.653	0.708	0.829		
VIQ	0.509	0.538	0.608	0.605	0.530	0.627	0.660	0.574	0.896	
VQ	0.799	0.591	0.472	0.645	0.491	0.750	0.561	0.590	0.610	0.905

BA, Blogger’s appearance; BP, Blogger professionalism; VQ, video quality; VIQ, video interaction quality; PV, perceived value; EC, expectation confirmation; P, pleasure; S, satisfaction; T, trust; CUI, continuous use intention. The diagonal is the square root of AVE.

Within the framework of this study, the assessment of the proposed model’s comprehensive quality was conducted using the goodness of fit (GOF) measure, as put forth by Tenenhaus et al. (99). The computation of the GOF is as follows:

$GOF=\sqrt{\bar{AVE}\times \bar{R^{2}}}$ = $\sqrt{0.767\times 0.587}$ =0.670.

**Figure 2:**
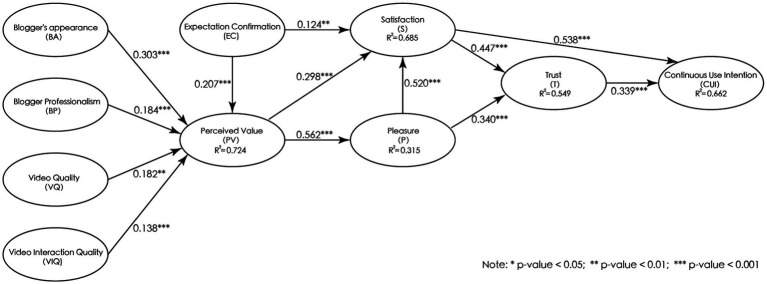
Hypothesis testing results. *p-value < 0.05, **p-value < 0.01, ***p-value < 0.001.

Based on the aforementioned outcomes, the GOF value was 0.670, surpassing the established threshold of 0.36 (100), proving the validity of the model. Furthermore, to validate the model according to the fit criteria proposed by Henseler et al. (101), an SRMA < 0.08 and NFI > 0.90 are considered indicative of good fit. Our analysis yielded an SRMR of 0.052 and an NFI of 0.833. Although the NFI is <0.90, it remains within an acceptable range. Additionally, Hair et al. (102), have emphasized the importance of the *R*^2^ value in evaluating the explanatory strength of the endogenous latent variables, which serves as a crucial reference for model fit. The higher *R*^2^ value indicates stronger predictive and explanatory power (97). In this study, The *R*^2^ values for the latent variables ranged from 0.315 to 0.724, indicating that the model possesses good fit. As shown in Figure 2. *Q*^2^ is utilized to assess the predictive ability of the model and serves as a metric for model fit as well (102). A *Q*^2^ value greater than 0 indicates predictive power, with higher values signifying stronger predictive capability. In this study, the *Q*^2^ values ranged form 0.216 to 0.558, thus supporting the conclusion that our model demonstrates good fit. In summary, the model in this study exhibits good fit, as evidenced by the GoF, SRMR, NFI, *R*^2^, and *Q*^2^ values.

### Hypothesis testing

In this study, PLS was employed to examine the path coefficients of the hypothesis model, reflecting the causal relationships and their strengths among the dependent variables. The *R* square values indicate the percentage of variance explained by the dependent variables and represent the predictive ability of the model. Bootstrapping was utilized to estimate the significance of each path coefficient. By resampling the data, the estimated values provide more accurate results than conventional asymptotic approximations (103). Thus, this approach was employed in this study to assess the significance of the relationships between variables.

In testing the hypotheses, it was found that image appearance, professionalism, video quality, and interaction quality of health science communication video bloggers positively influenced users’ perceived value. Specifically, the path coefficients and associated statistical values were as follows: (BA → PV, β = 0.303, *t*-value = 4.832; BP → PV, *β* = 0.184, *t*-value = 4.524; VQ → PV, *β* = 0.182, *t*-value = 3.324; VIQ → PV, β = 0.138, *t*-value = 3.134), supporting research hypotheses 1, 2, 3, and 4. In the context of examining the relationships among perceived value, satisfaction, and pleasure, it was demonstrated that perceived value positively influenced both satisfaction and pleasure. The following path coefficients and associated statistical values were observed: (PV → S, *β* = 0.298, *t*-value = 7.106; PV → P, *β* = 0.562, *t*-value = 15.228; P → S, *β* = 0.520, *t*-value = 11.844), supporting research hypotheses 5, 6 and 7. Regarding the hypotheses related to expectation confirmation, it was revealed that expectation confirmation positively influenced both perceived value and satisfaction. The path coefficients and associated statistical values were as follows: (EC → PV, *β* = 0.207, *t*-value = 4.668; EC → S, *β* = 0.124, *t*-value = 2.434), supporting research hypotheses 8 and 9. Examining the relationships among pleasure, satisfaction, and trust, it was found that pleasure and satisfaction positively influenced trust. The path coefficients and associated statistical values were as follows: (P → T, *β* = 0.340, *t*-value = 5.836; S → T, *β* = 0.447, *t*-value = 7.496), supporting research hypotheses 10 and 11. Finally, research hypotheses 12 and 13 were also supported, as it was observed that satisfaction positively influenced continuous usage intention (S → CUI, *β* = 0.538, *t*-value = 13.023). Additionally, trust positively influenced continuous usage intention (T → CUI, *β* = 0.339, *t*-value = 7.608). Thus, all the research hypotheses of this study were supported. The model’s path coefficients and significance levels are presented in Table 5 and Figure 2.

**Table 5:** Results of structural equation model.

Hypothesis	Path coefficient	*t*-value	Result
H1: BA →PV	0.303***	4.832	Supported
H2: BP →PV	0.184***	4.524	Supported
H3: VQ →PV	0.182**	3.324	Supported
H4: VIQ →PV	0.138**	3.134	Supported
H5: PV → S	0.298***	7.106	Supported
H6: PV →P	0.562***	15.228	Supported
H7: P →S	0.520***	11.844	Supported
H8: EC →PV	0.207***	4.668	Supported
H9: EC →S	0.124**	2.434	Supported
H10:P → T	0.340***	5.836	Supported
H11:S → T	0.447***	7.496	Supported
H12:S →CUI	0.538***	13.023	Supported
H13:T →CUI	0.339***	7.608	Supported

BA, Blogger’s appearance; BP, Blogger professionalism; VQ, video quality; VIQ, video interaction quality; PV, perceived value; EC, expectation confirmation; P, pleasure; S, satisfaction; T, trust; CUI, continuous use intention. **p-value < 0.01, ***p-value < 0.001.

### Testing of mediation effects

The Sobel test was used to determine the statistical significance of the proposed mediation model in this study. This involved obtaining *Z*-values that approximated the *p*-values. *Z*-value greater than 1.96 indicates the presence of a significant indirect effect between the independent and dependent variables (104). To further estimate the effects of the mediating variables, this study employed the bootstrap method with bias-corrected confidence estimates (105). Specifically, 95% confidence intervals for individual mediating effects were derived using 5,000 bootstrapped resampling iterations. If the value of 0 falls outside the 95% confidence interval, it indicates a statistically significant mediating effect, as shown in Table 6.

**Table 6:** Analysis of results of mediation effect.

Relationship	*z*-value of Sobel test	Path coefficient	Bias-corrected percentile bootstrap confidence intervals (95%)
BA→PV→ S	3.995***	0.090***	(0.052, 0.135)
BP→PV→ S	3.816***	0.055**	(0.014, 0.081)
VQ→PV→ S	3.010**	0.054**	(0.026, 0.093)
VIQ→PV→S	2.867**	0.041*	(0.014, 0.077)
BA→PV→P	4.605***	0.170***	(0.101, 0.233)
BP→PV→P	4.336***	0.103***	(0.057, 0.154)
VQ→PV→S	3.324**	0.054**	(0.026, 0.093)
VIQ→PV→P	3.069**	0.077**	(0.028, 0.130)
PV→P→T	9.349***	0.191***	(0.121, 0.274)
PV→S→T	5.157***	0.133***	(0.085, 0.194)
PV→S→CUI	6.237***	0.160***	(0.113, 0.215)
EC→PV→S	3.901***	0.062***	(0.036, 0.097)
EC→PV→P	4.463***	0.116***	(0.067, 0.175)
EC→S→T	2.315**	0.055*	(0.010, 0.116)
EC→S→CUI	2.392*	0.066*	(0.013, 0.124)
P→S→T	6.334***	0.233***	(0.177, 0.297)
P→S→CUI	8.762***	0.280***	(0.223, 0.343)
S→T→CUI	5.339***	0.152***	(0.106, 0.207)
P→T→CUI	4.630***	0.115***	(0.070, 0.176)

BA, Blogger’s appearance; BP, Blogger professionalism; VQ, video quality; VIQ, video interaction quality; PV, perceived value; EC, expectation confirmation; P, pleasure; S, satisfaction; T, trust; CUI, continuous use intention. *p-value < 0.05, **p-value < 0.01, ***p-value < 0.001. Number of bootstrap samples = 5,000.

## Discussion

This study used the C-A-C theory model to examine user perceptions of the content and form of health science popularization videos, with perceived value as a cognitive result. This study also considers expectancy confirmation and satisfaction in the ECT as mediating variables that influence users’ perceived value and continuous use intention. This study aimed to construct a model that identifies the factors affecting users’ continuous use intention of health science popularization videos by exploring the relationship among perceived value, expectancy confirmation, pleasure, satisfaction, trust, and continuous use intention.

### Relationship between health science popularization videos and perceived value

Perceived value, a result of users’ comprehensive evaluation of products (services), plays a key role in influencing users’ satisfaction, loyalty, and continuous use intention (56, 59). Typically, users are first influenced by factors such as the content and quality of products (services) during the usage process, which create the perceived value of the product. The level of this perceived value affects users’ emotions and subsequent behavior (62). The results of this study confirmed this process. The appearance, professionalism, information quality, and interaction quality of health science popularization video bloggers have a significant positive impact on users’ perceived value. This shows that, to improve people’s perceived value of health science popularization, it is necessary to comprehensively improve the content and format of health science popularization videos and satisfy users’ needs in terms of their visual, auditory, content, and experiential aspects.

### Relationship between expectation confirmation and perceived value and satisfaction

Expectation confirmation refers to the evaluation of user expectations after using an information system. During the process of continued information behavior, users will have expectations of the information system before using it. The perceptions generated during the use of the information system will be compared with the expected expectations. If the expectations are confirmed, it will lead to satisfaction and a willingness to continue to use it (36). This study confirmed that the confirmation of users’ expectations from health science popularization videos had a positive impact on perceived value and satisfaction. This is consistent with previous research results (65–67). This indicates that users’ expectation confirmation and perceived value are interdependent. To increase users’ satisfaction with health science popularization videos, it is necessary to ensure that users’ expectation confirmation and perceived value are consistent so that they can be satisfied and continue to use them.

### Relationship between emotions and continuous use intention

Pleasure, satisfaction, and trust reflect users’ psychological well-being, encompassing not only their emotional states during the utilization of products (or services) but also their influence on the intent for continued usage (69, 73, 74). This study substantiated that users’ pleasure and satisfaction were influenced by perceived value and, in turn, these feelings impacted users’ trust and intent for the continuous usage of health science communication videos. These findings are consistent with previously relevant research outcomes. Emotional changes constitute a multifaceted process influenced by numerous factors. In the context of users engaging in health science popularization videos, when their emotions lean toward pleasure, it leads to an elevation in satisfaction and trust, thereby deepening the genesis of their intention for continued usage. Thus, increasing people’s intention to continue using health science popularization videos involves cultivating positive emotional experiences throughout their utilization, thus reinforcing user satisfaction and trust in these videos.

### Implications

#### Implications for theory

This study amalgamated the C-A-C theoretical framework with the ECT to examine the underlying mechanisms of user intention for the continuous usage of health science popularization videos. It delves into the influences of the content and presentation factors of health science popularization videos on users’ perceived value, emotional states, and intent for continued usage. Furthermore, through the organic integration of these two theoretical models, existing models are enriched and expanded. This research revealed that the impact of health science popularization videos on continuous usage intention manifests through multiple pathways. It not only influences perceived value, satisfaction, and trust through the lens of expectation confirmation, thereby affecting continuous usage intention, but also directly influences continuous usage intention through perceived value, pleasure, satisfaction, and trust. Finally, this study refined the content and presentation factors of health science popularization videos, augmenting the realm of research in health science communication information and providing insights for the development of health science communication products.

#### Implications for practice

This study offers a fresh perspective on the development of health science communication. The appearance, professionalism, video information quality, and video interaction quality of health science communication video bloggers are pivotal factors that influence users’ perceived value and are crucial elements for enhancing the overall quality of health science popularization videos. Consequently, when developing new health science popularization videos, proactive adjustments to the appearance and demeanor of video bloggers should be made to align them with their professional characteristics. Displaying an image that exudes grace, sophistication, and a hint of liveliness not only captures users’ visual attention but also strengthens their sense of identification with the video blogger’s persona. Thus, showcasing the host’s professional background, experience, and qualifications is imperative.

In health science popularization videos, the portrayal of robust expertise and skills should be evident, along with a comprehensive exhibition of the bloggers’ professional competence to pique users’ interest. Lastly, in terms of information and interaction quality, the videos should offer easily digestible health science knowledge through a variety of formats, such as engaging storytelling, simplification, and concise presentation. This diverse approach serves to stimulate user interest and facilitate the comprehension and absorption of health information. Efforts should be made to refine the interactive communication between users and health bloggers and correctly guide users to access more health knowledge. Furthermore, enhancing subsequent health services and guidance is crucial as personalized guidance and services are delivered in a “one-to-one, point-to-point” manner. This approach ensures that every user receives high-quality health services and ongoing support, thus fostering sustained usage intent. By implementing such strategies, health science popularization videos can better serve the general public, thereby elevating the overall health literacy of the population.

#### Limitations and future directions

In this study, despite rigorous control over the research structure, methodology, and data collection, certain limitations remain that can be addressed in future research. First, the data sample used in this study was cross-sectional, which means that the analysis results can only explain the current relationship between health science popularization videos and individuals’ sustained usage intent. Therefore, residents’ long-term usage intent requires further observation over an extended period. Second, owing to limitations in time and space, the sample for this study was drawn exclusively from second- and third-tier cities. Therefore, they may not be representative of the entire population of Chinese residents. To address this, future research should encompass a broader range of cities and regions to enhance the accuracy of the study by collecting a larger sample size. Additionally, in subsequent studies, it would be valuable to examine whether resident characteristics (such as demographics, income, education level, and health awareness) influence their continued usage intention for mobile health science popularization videos.

## Conclusion

The findings of this study indicate that factors related to health science popularization videos (e.g., health influencer appearance, professionalism, video information quality, and video interaction quality) have a significant impact on users’ perceived value, emotions, and sustained usage intent. Government bodies, healthcare institutions, and media companies should collaborate to establish policies and manage communication efforts. This collaboration should aim to comprehensively enhance the quality of health science communication videos through authoritative, standardized, and widespread approaches. It is crucial for individuals to access reliable health science information. Additionally, such efforts can effectively contribute to increasing the overall health literacy level of the population.

## Data availability statement

The raw data supporting the conclusions of this article will be made available by the authors, without undue reservation.

## Author contributions

WX: Conceptualization, Funding acquisition, Investigation, Resources, Writing – original draft, Writing – review & editing. TP: Data curation, Methodology, Supervision, Validation, Writing – review & editing. LH: Data curation, Visualization, Writing – review & editing. KT: Data curation, Methodology, Project administration, Software, Writing – review & editing, Writing – original draft.
